# Crucial Role of miR-433 in Regulating Cardiac Fibrosis: Erratum

**DOI:** 10.7150/thno.63330

**Published:** 2021-06-08

**Authors:** Lichan Tao, Yihua Bei, Ping Chen, Zhiyong Lei, Siyi Fu, Haifeng Zhang, Jiahong Xu, Lin Che, Xiongwen Chen, Joost PG Sluijter, Saumya Das, Dragos Cretoiu, Bin Xu, Jiuchang Zhong, Junjie Xiao, Xinli Li

**Affiliations:** 1 Department of Cardiology, The First Affiliated Hospital of Nanjing Medical University, Nanjing 210029, China.; 2 Cardiac Regeneration and Ageing Lab, School of Life Science, Shanghai University, Shanghai 200444, China.; 3 Laboratory of Experimental Cardiology, University Medical Centre Utrecht, Utrecht 3508GA, The Netherlands.; 4 Department of Cardiology, Tongji Hospital, Tongji University School of Medicine, Shanghai 200065, China.; 5 Cardiovascular Research Center and Department of Physiology, Temple University School of Medicine, Philadelphia, PA 19140, USA.; 6 Cardiovascular Division of the Massachusetts General Hospital and Harvard Medical School, Boston, MA 02215, USA.; 7 Victor Babes National Institute of Pathology, Bucharest 050096, Romania.; 8 Division of Cellular and Molecular Biology and Histology, Carol Davila University of Medicine and Pharmacy, Bucharest 050474, Romania.; 9 Innovative Drug Research Center of Shanghai University, Shanghai 200444, China.; 10 State Key Laboratory of Medical Genomics & Shanghai Institute of Hypertension, Ruijin Hospital Affiliated to Shanghai Jiao Tong University School of Medicine, Shanghai 200025, China.

The authors regret that the original version of this paper 1 unfortunately contained some incorrect representative images of immunofluorescent stainings for cardiac fibroblasts. For presenting high resolution images, at the time of figure assembly, we reperformed *in vitro* cell experiments and took representative images under confocal microscope for EdU/α-SMA staining of cardiac fibroblasts. Meanwhile, we reperformed pHH3/Vimentin immunofluorescent staining of mice heart tissues. We apologize that at the time of figure assembly, we choose representative images by mistake. We confirm that it would not affect any results and conclusions of the paper. The correct representative images for Figure 4B, Figure 6B, Figure 9E, and Supplemental Figure 3C are shown below. The authors apologize for any inconvenience that these errors may have caused.

## Figures and Tables

**Figure 1:**
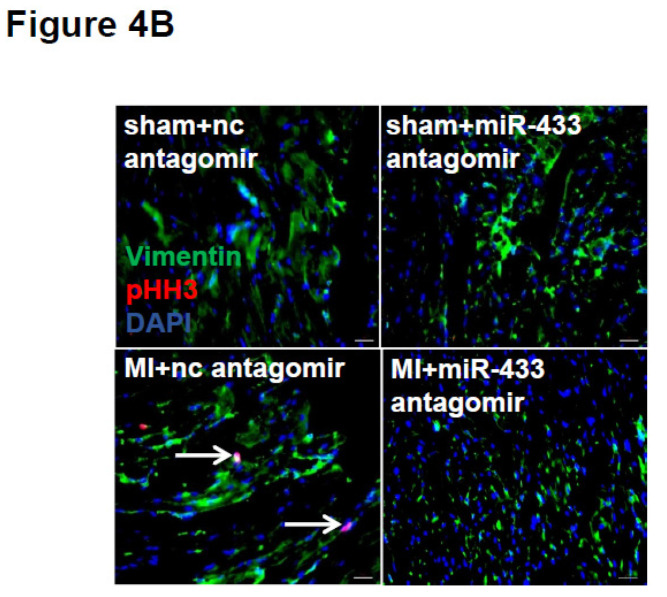
Corrected image for original Figure 4B.

**Figure 2:**
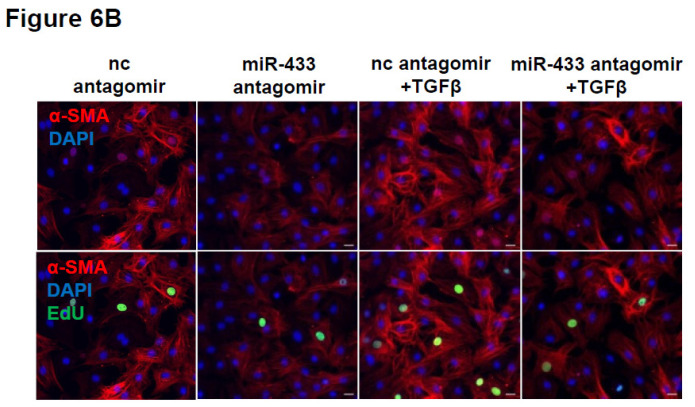
Corrected image for original Figure 6B.

**Figure 3:**
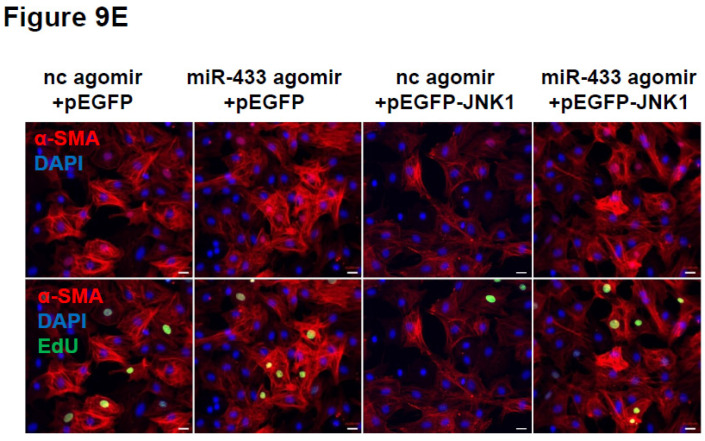
Corrected image for original Figure 9E.

**Figure 4:**
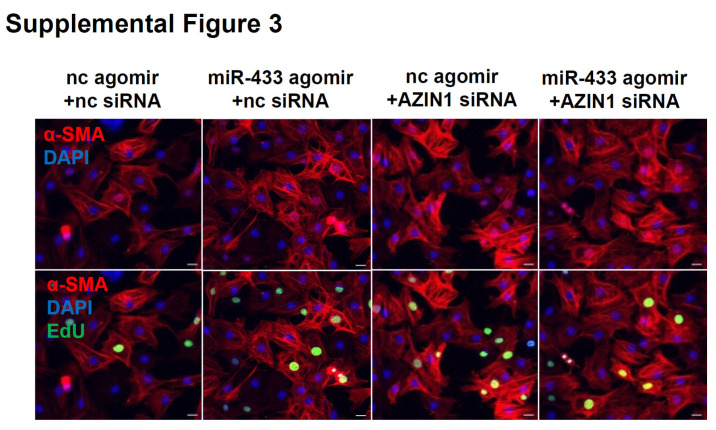
Corrected image for original Supplemental Figure 3.
